# Possibilities and limits of using gyroscopic sensors in the diagnosis of progression of osteoarthritis and femoroacetabular impingement syndrome

**DOI:** 10.1186/s13018-022-03141-1

**Published:** 2022-05-07

**Authors:** Pavel Holeka, Filip Studnička, Damián Bušovský, Jan Štěpán, Jan Matyska, Jan Šlégr

**Affiliations:** 1 Faculty of Medicine in Hradec Kralove, Charles University, Šimkova 870, 500 03 Hradec Králové, Czech Republic; 2 Department of Physics, Faculty of Science, University of Hradec Králové, Rokitanského 62, 500 03 Hradec Králové, Czech Republic; 3 Centre of Advanced Technologies, Faculty of Science, University of Hradec Králové, Rokitanského 62, 500 03 Hradec Králové, Czech Republic

## Abstract

Osteoarthritis is the most common type of degenerative joint disease and affects millions of people. In this paper, we propose a non-obtrusive and straightforward method to assess the progression of osteoarthritis. In standard medicine praxis, osteoarthritis is observed with X-rays. In this study, we use widely available wearable sensors with gyroscopes to make the observation. Two novel methods are proposed for gyroscope data processing. A small-scale study has shown that these methods can be used to monitor osteoarthritis’s progression, and to differentiate between healthy subjects and subjects with femoroacetabular impingement syndrome.

## Introduction

Osteoarthritis is the most common form of joint disease and it affects about 12% of the population [7]. Prevalence increases with age, and X-ray changes that are typical of osteoarthritis are found in almost 70% of people over 65 years of age. Osteoarthritis most often affects hip and knee joints but it is also commonly found in other locations (e.g., spine, shoulder, elbow, fingers, etc.).

Femoroacetabular impingement syndrome (FAI) is a motion-related condition with a complex morphology, symptoms and clinical signs [4, 12], which leads to osteoarthritis [2, 8]. The prevalence of FAI as a clinical diagnosis is estimated to be 10 to 15% in adult population [14].

While these diseases are commonly studied with X-rays (Fioruzzi et al. [6]), it would certainly be convenient to have a simple method of directly classifying the degree of severity in the doctor’s office. Wearable sensors that measure the mechanical properties of a person walking can be advantageously used to provide a solution to this problem. The aim of this paper is to present two possibilities of novel data processing using wearable sensors which may lead to measurement of progression of osteoarthritis and the presence of femoroacetabular impingement syndrome.

The implications of wearable devices in the healthcare industry are currently under rigorous study [5]. Studies show that wearable sensors are well-accepted and tolerated [22], and used together with other medical devices [9, 11, 15]. They are also used in many medical situations, such as in preventive health care [1, 10, 19]. The possibilities for self-monitoring of patients with age-related diseases look very promising [16, 17, 21].

## Materials and methods

For measurement, a wireless sensor was chosen because of the need to walk freely without any constraints. We used MBIENTLAB METAMOTION which consists of a wireless Nordic Semiconductor chip that transfers data over a 2.4 GHz Bluetooth network. This sensor comprises of accelerometer and gyroscope. To enhance the precision and achieve additional detail in signal data, a 3-axis gyroscope with a sensitivity of 125 degrees/second was chosen. The sampling frequency of transmitted data was 200 Hz. The data were collected using a cellphone with Android 9.0 and a proprietary application used for transferring the data into the cloud for further processing.

For experimental part one, four volunteers were chosen: one healthy male and female and one male and female, both suffering from femoroacetabular impingement syndrome (on the same, right side). The sensor was placed on the right side of the pelvis, and each volunteer walked for 15 min on a treadmill.

For the second part of the experiment, 62 patients were selected. The inclusion criteria were indication to arthroscopy based on examination by physician and confirmed osteoarthritis stage based on X-ray imaging, and the exclusion criteria were morbid obesity (body mass index larger than 35) and disability to finish walking test because of the need of crutches to walk. Among them were 35 males and 27 females, the average age was 41 ± 12, and average weight was 74 ± 13 kg. In total, 19 patients had stage 1 osteoarthritis, 31 patients had stage 2 arthrosis, and 12 patients had stage 3 arthrosis on the grading scale for plain radiographs of the hip [20]. The sensor was placed on the right-hand side of the pelvis, and the patients walked approximately 10 steps. Three axes of the gyroscope signal were recorded as a time series, with a sampling frequency of 200 Hz.

A novel approach to signal processing was proposed to evaluate the risk of osteoarthritis. Osteoarthritis is often connected with crunching and grinding emerging from moving joints [13]. These mechanical disturbances are based on obstructions on mechanical movement and are often called crepitus. This grinding as a mechanical noise is then propagated through the body into the motion disturbances of the sensor which is sensitive enough to capture this mechanical noise while the patient is walking. To compare patients with different stages of osteoarthritis, it is first necessary to normalize the analyzed signal to eliminate any discrepancies, such as other types of pace. To achieve this, an approach using differential geometry was chosen. We treat three signals as time series of projections of the measured phenomena (pace) to three orthogonal axes. Euclidean differential geometry invariant, called arc length, was then used as a descriptor of the walking pace. Arc length is a mathematical object that is invariant under the action of the group ${\text{SO}}\left( 3 \right) \to {\text{R}}^{3}$, so it is invariant under translation and rotation in Euclidean space. This means that the resulting studied quantity (i.e., the arc length) amplifies small changes in the measured signals, which should be connected with crepitus, while maintaining and simplifying the information about individual steps. The arc length is calculated as follows:

$$s\left( t \right) = \mathop \smallint \limits_{0}^{t} \sqrt {\mathop \sum \limits_{i = 1}^{3} \left( {\frac{{{\text{d}}G_{i} \left( \tau \right)}}{{{\text{d}}\tau }}} \right)^{2} } {\text{d}}\tau ,$$

where $G_{i}$ is the *i*-th signal from gyroscope and $\tau$ is sampling time. To eliminate the natural linear trend of arc length, the derivative of $s\left( t \right)$ was used as an input to the next part of the signal processing. The derivative of arc length (DAL) was calculated as a difference between two adjacent samples in each sampling time. Arc length approach has already been proven to be a strong tool in biosignal processing [3, 18].

To evaluate the emergence of crepitus, spectral properties of the derivative of arc length were studied. We present the results of two hypotheses, which we propose in the presented paper.

The first hypothesis claims that with an increased stage of osteoarthritis, more parasitic mechanical noise should emerge in the frequency spectra of the processed signals. To further eliminate any disturbances, the derivative of arc length was decomposed to 2-s intervals, on which the Welch spectral analysis was performed. The resulting spectra are power spectral densities of the signals, where these spectral densities were calculated using Welch’s method. All of the measured spectra for each individual in these 2-s intervals were averaged. This ensures that minor local disturbances will not project into resulting spectral characteristics. After calculating the spectra for each patient, the resulting spectra for each stage of osteoarthritis were averaged. Consequently, the final result was three double-averaged spectral properties, each belonging to one stage of osteoarthritis.

The second hypothesis also takes the derivative of arc length as the input signal to represent the risk of osteoarthritis and the presence of crepitus. However, instead of Welch spectral analysis, continuous wavelet transformation (CWT) was used to analyze the spectral properties. CWT is effective at studying the low-frequency band of the spectra. We hypothesize that in the case of low risk of osteoarthritis, the pace and motion should be fluent without any discrepancies generated by crepitus. This means that the spectra in the higher part of the measured spectrum should contain all of the frequencies with various magnitudes without any significant frequency drops, similar to white noise. However, in the presence of crepitus, there should be visible drops in higher frequency spectra generated by spikes of mechanical noise generated by crepitus. This means that there are some resonances present. To analyze this, we first calculate the CWT of DAL. We then split the result of CWT into individual footsteps of measured subjects, and we average the CWT spectra over these footsteps. Finally, we take the average of these spectra over all subjects in each of the three groups with different stages of clinically confirmed osteoarthritis and compare them.

## Results

### Experiment part one: cohort of *N* = 4 volunteers

**Fig. 1:**
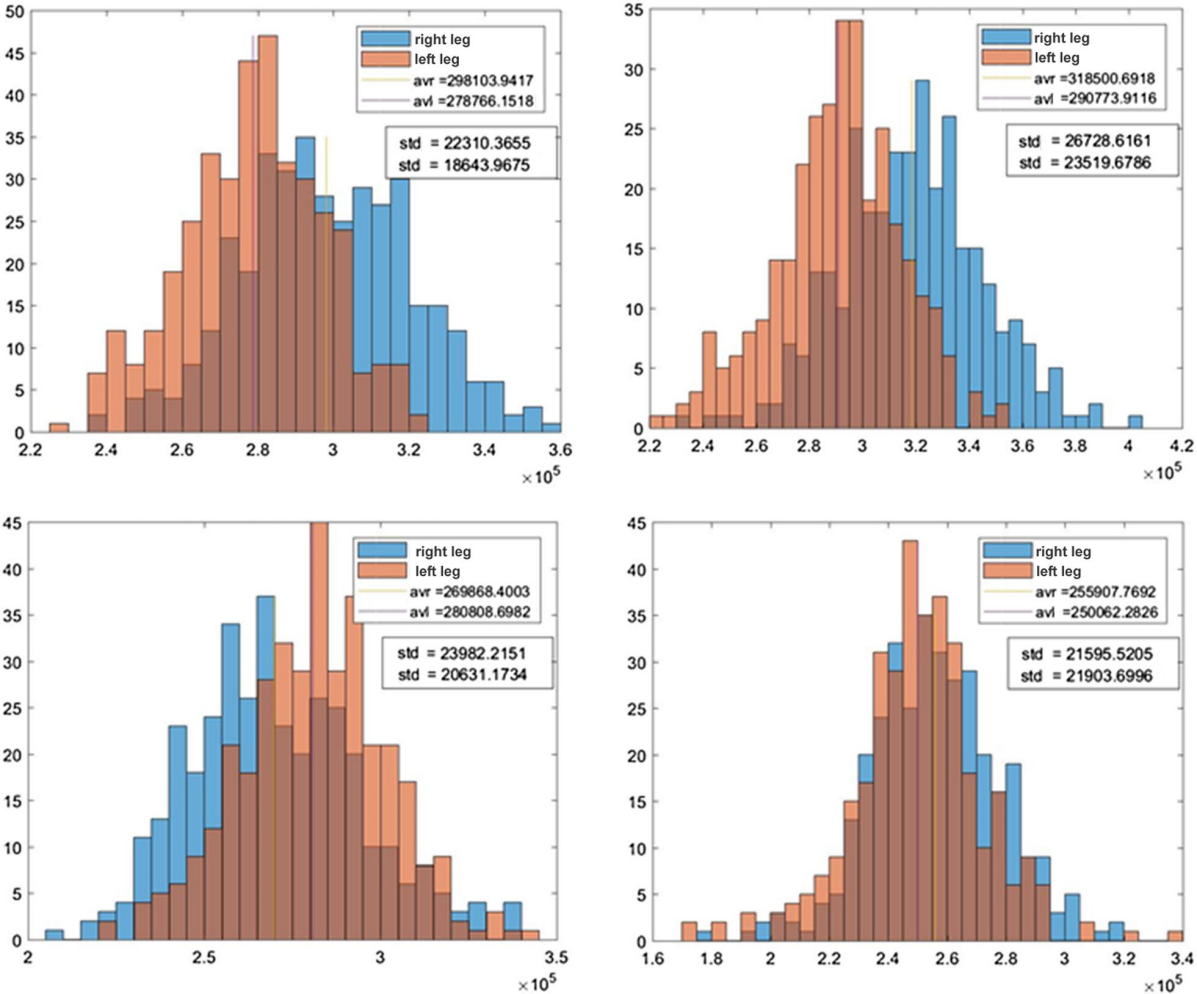
Histograms of left and right leg time series differences

A 3-axis gyroscope with a sensitivity of 125 degrees/second was chosen to enhance the precision and achieve additional detail in the signal data. The time distances between consecutive steps were analyzed. Only arc length with subtraction of linear trend was used, so the principal part of the signal consists of individual steps. The maxima in the signal correspond with the movement of the right leg and the minima with the left leg. By finding these minima and maxima, two time series can be obtained, where the first corresponds to the time distance of the right leg step and the second corresponds to the left leg step. By statistical analysis of these time series, it is possible to find discrepancies between left and right leg movement, which by hypothesis should differentiate between healthy volunteers and volunteers with femoroacetabular impingement syndrome because one of the manifestations of this syndrome is limp leg. At the same time, this may not be visually apparent, especially in the early stages of the disease. To compare the results, histograms of left and right leg time series differences were used (see Fig. 1).

The top left-hand and top right-hand histograms correspond to volunteers suffering from femoroacetabular impingement syndrome, the bottom left-hand histogram was obtained from data with a healthy volunteer who limped using sharp object placed in his right shoe, while the bottom right histogram was obtained from data with a healthy volunteer. It can be seen that in bottom right-hand histogram that the left and right legs have similar statistical properties, unlike in the other histograms where the left and right legs differ significantly.

### Experiment part two: cohort of *N* = 62 patients

**Fig. 2:**
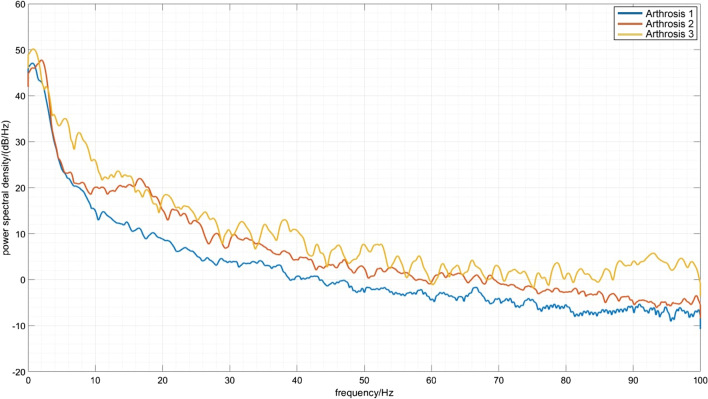
Three evaluated spectra of different stages of arthritis corresponding to possible crepitus

For the first hypothesis, three evaluated spectra were studied for possible changes correlated with crepitus and the stage of osteoarthritis. The resulting spectra are given in Fig. 2.

**Fig. 3:**
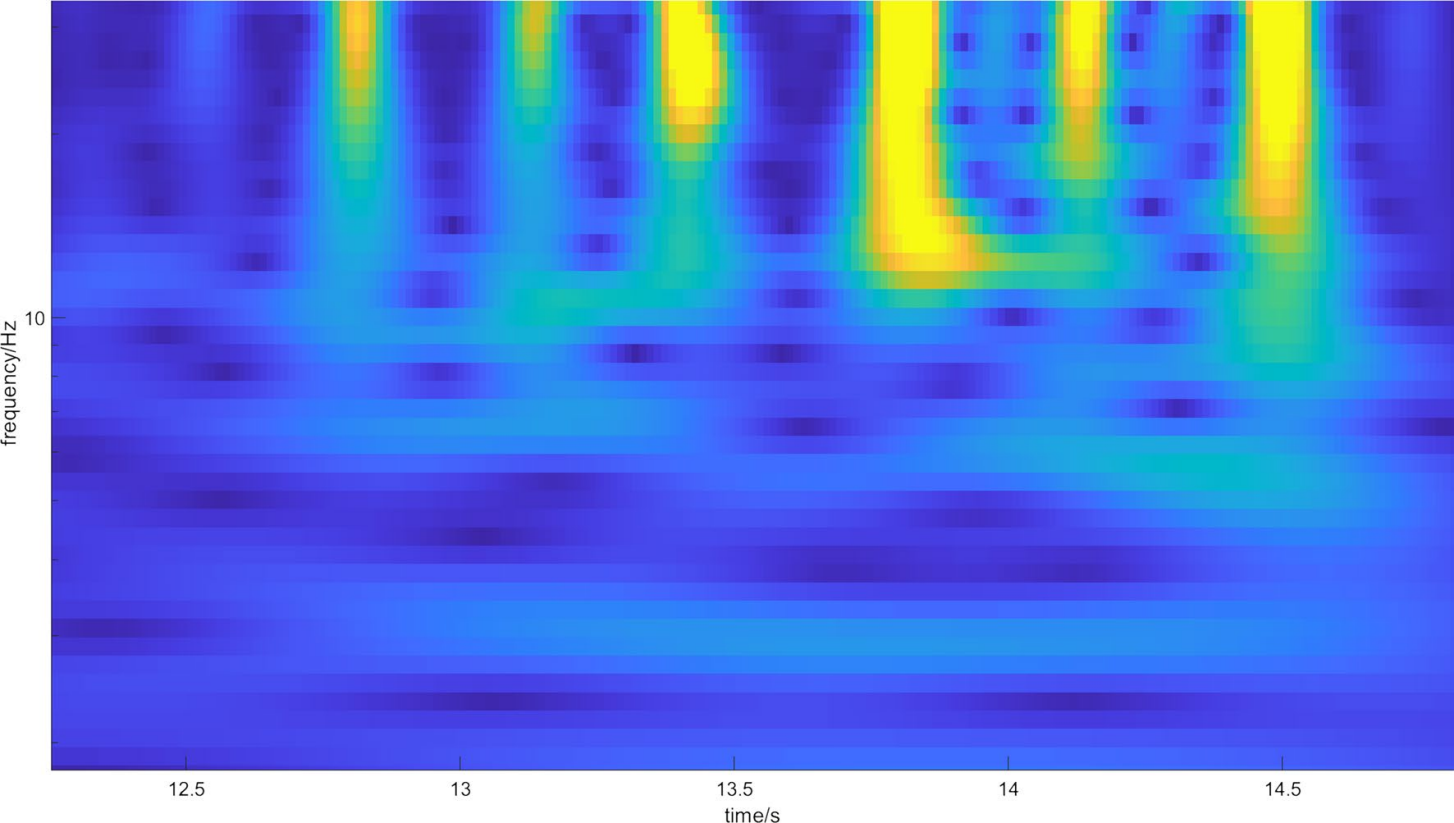
Magnitude scalogram of CWT of seven footsteps of one subject with arthritis stage 1. The magnitude is represented by the color, where blue represents low magnitude and yellow represents high magnitude. All of the steps propagate along the spectrum, with few resonances represented by blue dots (minima of the spectrum)

**Fig. 4:**
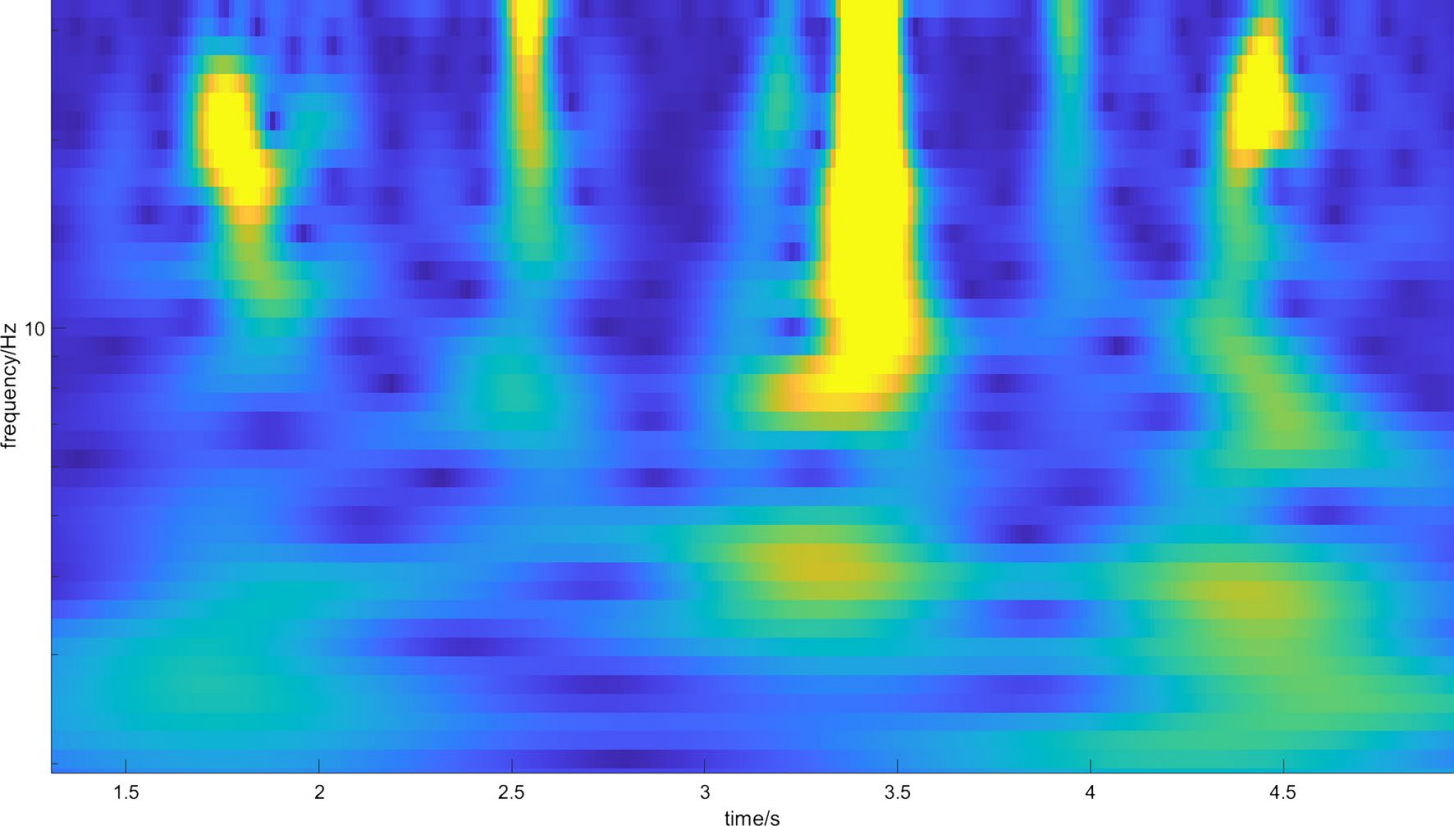
Magnitude scalogram of CWT of five footsteps of one subject with arthritis stage 2. The magnitude is represented by the color, where blue represents low magnitude and yellow represents high magnitude. More blue dots (minima of the spectrum) are emerging in the spectrum, which represent emerging resonances caused by crepitus, but it is still difficult to distinguish them from stage 1 arthritis

**Fig. 5:**
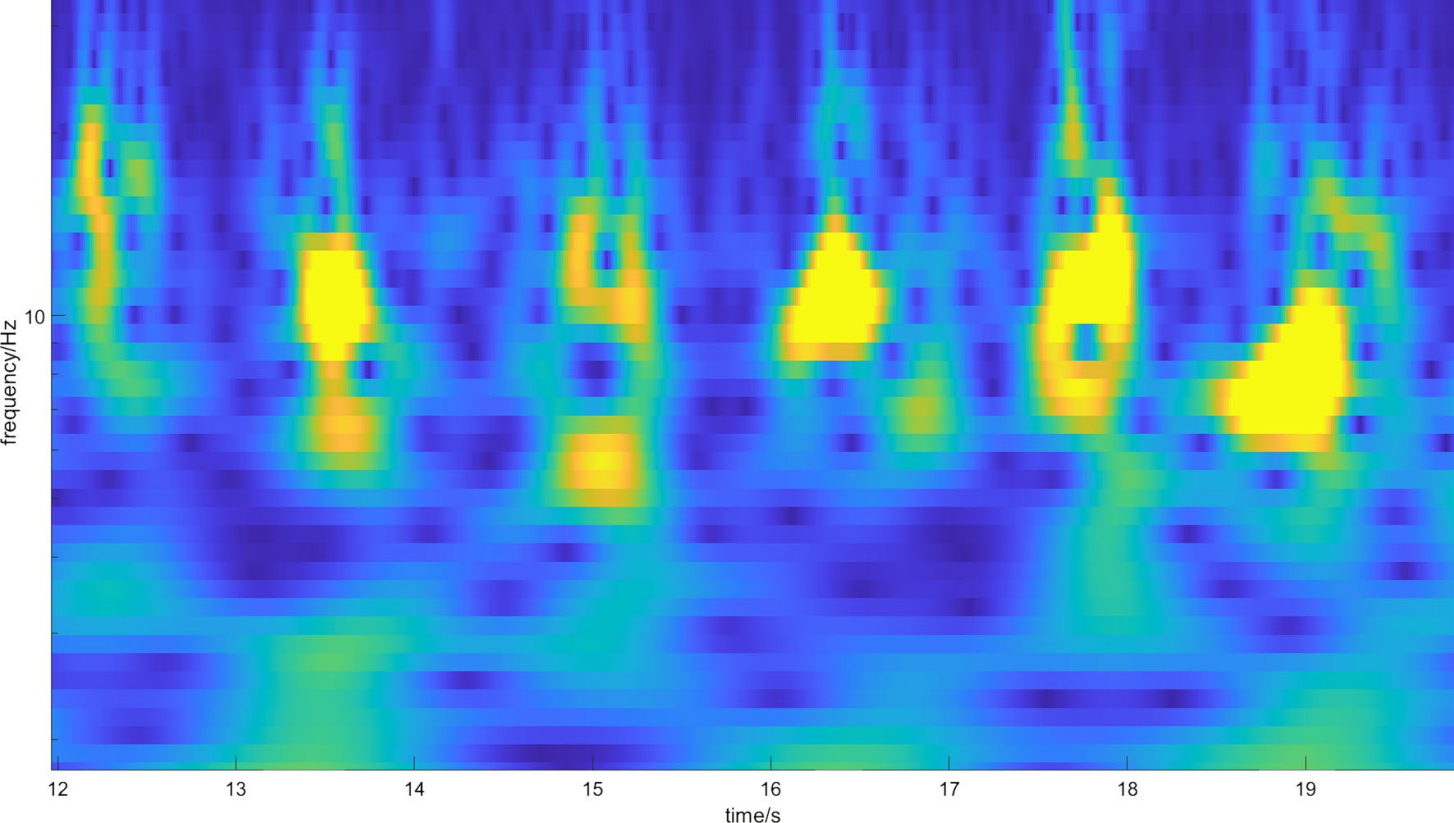
Magnitude scalogram of CWT of six footsteps of one subject with arthritis stage 3. The magnitude is represented by the color, where blue represents low magnitude and yellow represents high magnitude. Individual steps are composed nearly only from blue dots (minima of the spectrum). There are many resonances in the spectrum caused by crepitus

All of the spectra have similar properties in the low-frequency range. The arthritis stage one spectra do not manifest any significant changes in higher frequency ranges. This is plausible given that stage one arthritis does not manifest changes in the roughness of touching cartilage in the joints, so no additional mechanical frequency disturbances should be present. Arthritis stage two shows increased power spectral density in the 18 Hz range, indicating an increase in mechanical frequency disturbances that are probably generated by increased friction in the joints. Arthritis stage three shows an increase in power spectral density even sooner from the 5 Hz range and an increase in the 90 Hz range, indicating more mechanical frequency disturbances. To check the statistically significant difference between the spectra, we have calculated two paired two-sample t tests between spectra for the first and second stage of arthritis, and the first and third stage of arthritis. This is possible thanks to the method that was used to calculate the spectra (i.e., as a double average of multiple single spectra). Hence, it is possible to apply the central limit theorem. The results of test statistics are $t_{12} = 243$ and $t_{13} = 412$, which are well inside the critical region. For the second hypothesis, we calculated CWT for all subjects, and then averaged them over individual footsteps and over the groups with the same stage of osteoarthritis. Typical CWT spectra for each stage of osteoarthritis are given in Figs. 3, 4, 5.

**Fig. 6:**
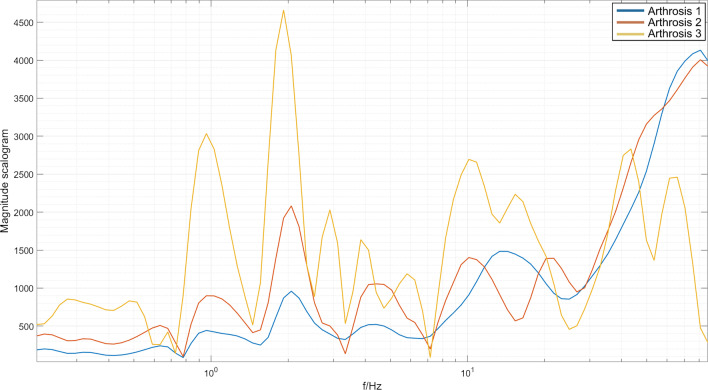
Magnitude scalogram of averaged spectra for all steps in each stage of arthrosis

It is now possible to establish a threshold in CWT magnitude to find each footstep. The threshold was found empirically: we have integrated the spectrum for each column of CWT (such as in Fig. 3) and neglected the parts of spectra with no footsteps below the threshold of magnitude. All of the spectra were then averaged over the individual groups of different stages of osteoarthritis. The result is given in Fig. 6. The idea now is to calculate the number of local minima, which corresponds with the mechanical filtering of the signal caused by crepitus. The number of minima corresponding to each stage of arthritis is: Arthritis stage 1–7 minima; Arthritis stage 2–8 minima; Arthritis stage 3–11 minima. This implies that there is a possible correlation between the number of disturbances in the magnitude spectrogram and the stage of arthritis. This causation is strengthened by the fact that crepitus causes mechanical disturbances, which should be represented in the frequency spectrum.

## Discussion

Three novel methods were used to analyze pathologies in human pace. The first method used arc-length parametrization to extract information about the time differences between consecutive leg steps. It was found that it is possible to show differences between the movement of left leg and right leg by using arc-length parametrization to extract time differences. This method can be used to check the lateral imbalance of pace for people after injuries of (for example) ligaments in the knees or to check whether their rehabilitation after injury is being performed correctly. This method can also be used to check the early stages of femoroacetabular impingement syndrome, which expresses as mild limp.

The second and third methods were used to distinguish between different stages of arthritis. The second method focused on finding the emergence of crepitus using spectral analysis. The results were not very conclusive, and so a third method was proposed where the spectral properties of arc-length parameterization of the signal were analyzed using CWT. This method showed very promising results. This method should also stimulate further research in this area because it can be a very useful tool to monitor the progress of arthritis using cheap wearable sensors. Consequently, the medication for cartilage repair can be prescribed earlier, which will slow the progression of arthritis.

## Conclusions

Our initial results show that novel approaches in signal processing of gyroscope signals used to detect arthritis stage and femoroacetabular impingement syndrome could lead to more robust detection using a wearable biosensor system. Three methods were proposed and tested on real data with promising results, showing a correlation between pathologies of the human locomotor system and resulting calculations. The aim of this work is to propose these new approaches and to encourage further work in this area. At the same time, all of the results of this study were further confirmed by a medical professional based on X-ray imaging.
